# The Recent Progress of Pitch Nanoengineering to Obtain the Carbon Anode for High-Performance Sodium Ion Batteries

**DOI:** 10.3390/ma16134871

**Published:** 2023-07-07

**Authors:** Wen-Sheng Du, Chen Sun, Qiang Sun

**Affiliations:** School of Metallurgy, Northeastern University, Shenyang 110819, China; dws21enuer@163.com (W.-S.D.); sc18641951120@163.com (C.S.)

**Keywords:** sodium ion battery, anode, pitch-based carbon

## Abstract

As an anode material for sodium ion batteries (SIBs), carbon materials have attracted people’s interest because of their abundant resources, good structural stability and low cost. Among most carbon precursors, pitch is viewed as a promising one because of a higher carbon content, good oxidation reversibility and low cost. However, the pitch-based carbon obtained with direct pyrolysis of pitch displays a high degree of graphitization and small layer spacing, which is unfavorable for the storage of sodium ions. In recent years, with the aid of the development of the nanoengineering process, the storage of sodium ions with pitch-based carbon has been drastically improved. This review article summarizes the recent progress of pitch nanoengineering to obtain the carbon anode for high-performance SIBs, including porous structure adjustment, heteroatom doping, co-carbonization and pre-oxidation. In addition, the merits and demerits of a variety of nanoengineering processes are discussed, and future research directions of pitch-based carbon are prospected.

## 1. Introduction

As the demand for the replacement of traditional fossil fuels by renewable energy and related large-scale energy storage systems (EES) has continued to increase, battery technologies and applications have been developed rapidly [1]. Lithium ion batteries (LIBs) have developed rapidly in the portable electronic market and expanded to EES applications due to their high energy density, no memory effect and long life [2,3]. Nevertheless, lithium is low in the earth’s crust, and large-scale demand forces us to seek new alternatives [4]. Because sodium ions have similar physicochemical properties with lithium ions and higher crustal reserves, in renewable energy storage systems, sodium ion storage is a powerful alternative to lithium ion storage [5].

However, sodium ions have a higher electrochemical potential relative to lithium ions (−2.71 V Na/Na^+^ and −3.04 V Li/Li^+^ vs. SHE) and higher ion radius (Na^+^/1.02 Å; Li^+^/0.76 Å), leading to slow adsorption/insertion kinetics and a lower energy density for SIBs [6]. Furthermore, because of large sodium ions and thermodynamic instability of sodium–graphite composites, the graphite used as the anode in commercial LIBs cannot be directly used in SIBs [7]. Hence, the key to the development of SIBs is the suitable anode materials [8].

Currently known anode materials for SIBs include carbon-based materials, alloys, metal oxides, nanosheet structure, organic composites, etc. [9,10,11]. Among them, carbonaceous materials are favored because of their structural stability, abundant resources, low cost and feasibility for practical applications [12,13]. Hard carbon obtained with carbonization of a biomass or polymer has large interlayer spacing and a disordered structure, which is conducive for Na ion insertion/extraction [14,15]. In 2000, the Na^+^ storage capacity of hard carbon typically reached a value of 300 mA h g^−1^ firstly proposed by Dahn and Stevens [16]. The mechanism of sodium ion storage in hard carbon can be divided into three different processes including (1) sodium adsorption on the surface and the defect, (2) sodium insertion between carbon layers, (3) sodium filling inside the closed nanopores. However, the defects in the disordered structure of hard carbons would induce a high surface area, which could cause a low initial coulomb efficiency (ICE). In addition, the lower degree graphitization of hard carbon led to a low conductivity, which resulted in a poor rate performance. In addition, due to the relatively low carbon yield and high cost, hard carbon is limited in its industrial application in SIBs [17,18,19]. In contrast, soft carbon with relatively ordered carbon layers and a high graphitization showed a higher conductivity [20]. In contrast to hard carbon, soft carbon with a relatively high graphitization of small interlayer spacing showed a higher conductivity [21].

Pitch, which is a byproduct in petroleum and coal industries, has a high carbonization yield, high polycyclic aromatic hydrocarbon content and abundant resources, and is considered as a promising candidate for carbonaceous anode synthesis for SIBs [17,22,23]; pitch contains a large number of sp^2^ structures, and the carbon material obtained with direct pyrolysis of pitch has a poor sodium storage performance due to its high degree of graphitization and small interlayer spacing [24,25].

A previous study demonstrated that sodium could overcome the energy barrier and insert into the carbon layer when the interlayer spacing is larger than 0.37 nm [26]. Furthermore, the defects and the pores were also proved to be efficient sites for sodium storge. Up until now, researchers have devoted most of their energy to obtaining pitch-based carbon materials with a high electrochemical sodium storage performance based on nano-engineering processes. Also, some researchers have contributed to the review works on the application of pitch-based carbons for SIB anodes in the context of carbon’s morphology and structure [18]; while differences form most review works, this paper summarizes the typical synthetic strategies for pitch-based carbon materials for SIBs, such as porous structure adjustment, heteroatom doping, co-carbonization and pre-oxidation, which can fill a critical gap in the field. At the same time, we pay more attention to the influence of some element content changes on pitch composite carbon. Finally, a future trend in pitch-based carbon development is prospected.

## 2. Nanoengineering of Pitch for High-Performance SIB Carbon Anode

### 2.1. Porous Structure Adjustment

The establishment of a porous structure is an efficient strategy for improving the sodium storage performance of carbon; the porous structure can not only shorten the diffusion path of the ions but also make the penetration of the electrolyte easier [27]. As for pitch-based carbon, template carbonization is a common method for adjusting the porous structure [28,29,30].

Using nano-CaCO_3_ as a template, porous soft carbon (MSC) was fabricated from the mesophase pitch [31]. Compared to MPC (without the nano-CaCO_3_ template) with a non-porous structure, MSCs showed a more developed mesoporous structure and more disorder, which demonstrated that nano-CaCO_3_ templates hinder the development of ordered graphitic structures. Due to the use of the nano-CaCO_3_ template, MSC has a higher specific surface area (113.1 m^2^ g^−1^) than MPC (3.5 m^2^ g^−1^). MSC delivered an initial reversible capacity of 331 mAh g^−1^ at the current density of 30 mA g^−1^. In comparison, the MPC had a capacity of only 243 mAh g^−1^. When testing at a high current rate, the MSC electrode had a reversible capacity of 103 mAh g^−1^ after 3000 cycles at 500 mA g^−1^. However, due to the large specific surface area of MSC, ICE of MSC was only 45%, lower than that of MPC (71%).

**Figure 1:**
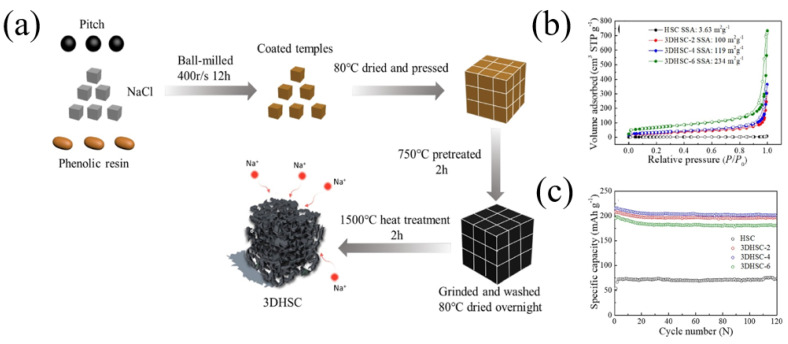
(**a**) Schematic illustration of the fabrication process of 3DHSC; (**b**) nitrogen sorption isotherms of HSC and 3DHSCs; (**c**) cyclic performance of HSC and 3DHSCs at a current density of 0.05 A g^−1^ [33] (Copyright 2019, Elsevier B.V. and Science Press).

Through assistance with a NaCl template, Lu et al. [32] prepared three-dimensional amorphous carbon (3DAC) with low-cost pitch and phenolic resin. Compared with the AC (without the NaCl template) with a specific surface area of 10.8 m^2^ g^−1^, the specific surface area of the 3DAC increased to 32.8 m^2^ g^−1^ because the NaCl template could introduce mesopores and macropores into the 3DAC. Therefore, the 3DAC sample delivered a high reversible capacity of 280.1 mAh g^−1^ at the current density of 30 mA g^−1^, and the ICE was 75%. Similarly, through assistance with a NaCl template, Qiu et al. [33] prepared three-dimensional pitch composite carbon (3DHSC) with phenolic resin and pitch (Figure 1a). The porous structure could be precisely regulated with a different mass ratio of NaCl and carbon precursors (mass ratio—2:1, 4:1, 6:1). With an increase in the NaCl template, carbon materials can introduce micropores, mesopores and macropores. The specific surface areas of HSC (without the NaCl template), 3DHSC-2, 3DHSC-4 and 3DHSC-6 (mass ratio—2:1, 4:1, 6:1) were 3.63, 100, 119 and 234 m^2^ g^−1^, respectively (Figure 1b). The results showed that 3DHSC-4 (mass ratio—4:1) delivered a high reversible capacity of 215 mAh g^−1^ at the current density of 50 mA g^−1^, and the ICE was 60%. In addition, the 3DHSC-4 electrode had a good cycle stability, with a reversible capacity of 200.7 mAh g^−1^ after 120 cycles at 50 mA g^−1^ (Figure 1c).

The microstructure has a significant effect on the electrochemical performance of carbon materials for SIBs. A suitable porous structure can effectively improve the sodium ion storage capacity of pitch-based materials. The porous structure can provide more reversible Na^+^ storage active sites and shorten the Na^+^ diffusion path, thereby improving the electrochemical performance of pitch-based carbon materials [34]. However, a large specific surface area could lead to a higher electrolyte consumption, resulting in lower ICE [35]. Therefore, how to adjust the appropriate porous structure to achieve pitch-based carbon materials with high ICE and a good electrochemical performance is still a challenging task.

### 2.2. Heteroatom Doping

Heteroatom (B, N, O, S and P) doping is usually used to modify carbon materials, which can create some defects to enhance the sodium storage capacity. Otherwise, the doped heteroatoms can also increase the conductivity and adjust the d space of the carbon interlayer [36]. Therefore, heteroatom doping has been widely used to optimize the structure of pitch-derived carbon to enhance the electrochemical performance when used as anodes in SIBs.

**Figure 2:**
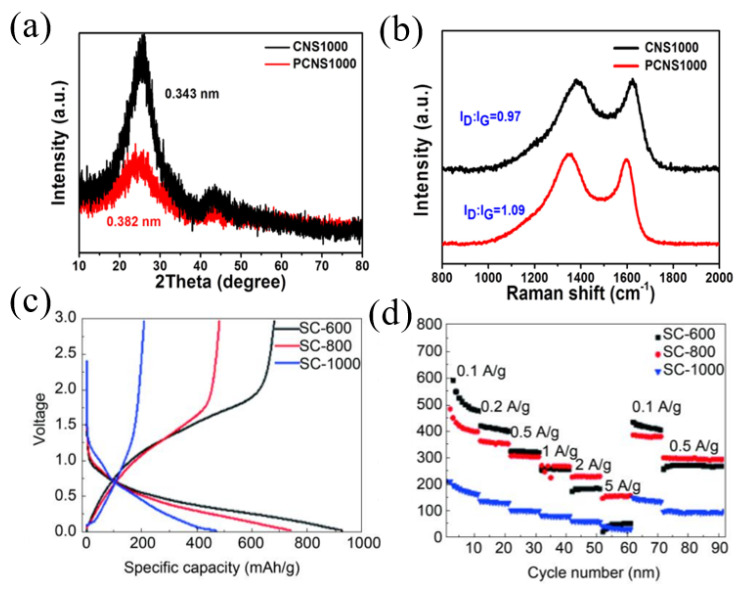
(**a**) XRD patterns of CNS1000 and PCNS1000; (**b**) long-term cycling stability measurement of PCNS1000 [41] (Copyright 2018, Elsevier B.V). (**c**) The first charging and discharging curves of SC600, SC800 and SC1000; (**d**) rate performance measurement of SC600, SC800 and SC1000 [7] (Copyright 2020, Elsevier Limited).

Nitrogen-doped carbons have been extensively studied because N-C bonds are relatively easy to form. In addition, the doping of nitrogen with carbon materials can not only effectively increase the electronic conductivity but also supply sufficient active sites to enhance the electrochemical performance for SIBs [26,37]. In general, nitrogen doping with carbon materials exists in three states including pyridinic nitrogen, pyrrolic nitrogen and graphitic nitrogen. Theoretical studies have shown that pyridinic nitrogen and pyrrolic nitrogen working as defects can provide more active sites for sodium storage, and graphitic nitrogen provides lone pair electrons to enhance electronic conductivity of pitch-derived carbon [38,39,40]. Hao et al. [41] prepared N-doped carbon nanosheets using coal tar pitch as a raw material and an NaCl template process followed by NH_3_ treatment. The N content increased from 1.10 wt.% to 4.17 wt.% compared to the carbon material without NH_3_ treatment, demonstrating the doping effect of NH_3_ treatment. The layer spacing of N-doped carbon nanosheets was 0.382 nm, which was much larger than the average layer spacing of carbon material without NH_3_ treatment (0.343 nm) and graphite (0.335 nm), and the structure was more disordered (Figure 2a). Therefore, the nitrogen-doped carbon nanosheets showed a discharge capacity of 302 mAh g^−1^ at a current density of 0.1 A g^−1^, then stabilized at a value of 270 mAh g^−1^. Even cycled at higher current densities of 0.2, 0.5, 1, 2 and 5 A g^−1^, high capacities of 228, 186, 167, 150 and 133 mAh g^−1^ could still be retained, which were all higher than that of the carbon material without NH_3_ treatment. In particular, even at an ultra-high discharge current density of 10 A g^−1^, the PCNS1000 still remained at a capacity of 124 mAh g^−1^, corresponding to a capacity retention of about 46% (Figure 2b). However, due to the large specific surface area of PCNS1000 (546 m^2^ g^−1^), the ICE was only 66%. Xue and co-workers [42] synthesized nitrogen-doped carbon using melamine foam as a template, coal pitch as precursors and the assistance of ferric nitrate nonahydrate, which delivered a stable cyclability, showing the high reversible storage capacity of 111 mAh g^−1^ at a current density of 3 A g^−1^ after 1000 cycles.

Besides nitrogen, sulfur is also usually used as a doping atom to expand the interlayer spacing of pitch-derived carbon as sulfur has a much larger relative atomic radius. At the same time, sulfur can also provide additional active sites for sodium storage to improve electrode kinetics [43,44,45]. For example, He et al. [7] reported a preparation of sulfur-doped pitch-based carbon materials using medium-temperature coal tar pitch and sublimated sulfur as starting agents through two-step carbonization processes. With an increase in the carbonization temperature, the content of sulfur decreased from 28.02 wt.% (600 °C, SC600) to 20.19 wt.% (800 °C, SC800), and finally to 8.64 wt.% at a temperature of 1000 °C (SC1000). However, with an increase in the carbonization temperature, the interlayer spacing of the pitch-derived carbon was increased from 0.358 nm (600 °C, SC600) to 0.368 (800 °C, SC800) and 0.380 nm (1000 °C, SC1000). When used in SIBs, the SC-600 sample demonstrated a better electrochemical performance (Figure 2c), with a discharge/charge capacity of 927.7/682.1 mAh g^−1^, higher than that of SC-800 (740.9/482.8 mAh g^−1^) and SC-1000 (471.0/209.5 mAh g^−1^). For rate performance measurement, SC-600 had the highest specific capacity among the three samples at current densities of 0.1, 0.2 and 0.5 A g^−1^. When the current density was set higher than 0.5 A g^−1^, SC-800 showed the highest specific capacity compared with the other two samples (Figure 2d).

**Figure 3:**
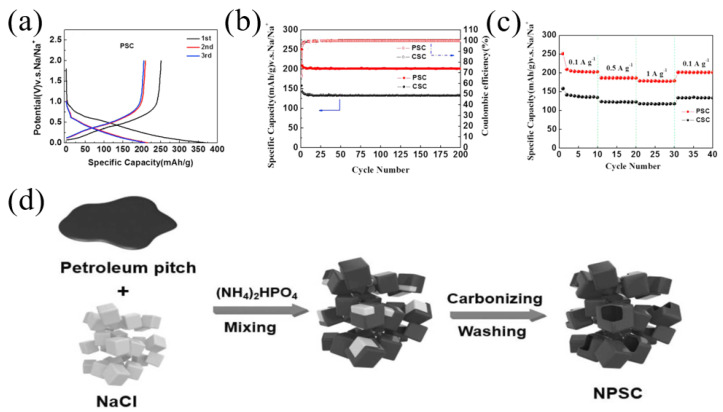
(**a**) The charge/discharge curves of PSC; (**b**) cycling performance at 100 mA g^−1^; (**c**) rate performance [48] (Copyright 2016, Elsevier B.V). (**d**) Illustration of material preparation [50] (Copyright 2022, Elsevier B.V).

Phosphorus has a low electronegativity and strong electron giving ability, and carbon with P doping almost shows significantly increased interlayer spacing so that the electrochemical performance of carbon materials can be improved [46,47]. For example, Sun et al. [47] prepared a phosphorus-doped pitch-derived carbon nanosheet using low-temperature pitch as a raw material and sodium hypophosphite as a phosphorus source; the obtained carbon delivered an enhanced electrochemical performance with high-rate capacity and long-term cycling stability, showing a capacity of 189 mAh g^−1^ at 2 A g^−1^ after 500 cycles, as well as an extraordinarily high capacity of 80 mAh g^−1^ at 20 A g^−1^ after 10,000 cycles. Miao et al. [48] fabricated phosphorus-doped pitch-derived carbon material (PSC) with pyrolysis of the coal tar pitch and H_3_PO_4_. XPS measurement indicated that the P content of the obtained carbon was 3.42 wt.% and the main chemical state of the phosphorus element was attributed to P-C, P-O-C and P-C-O bonds. The PSC electrode showed a higher capacity of 251 mAh g^−1^ than 158 mAh g^−1^ of CSC (without P doping) (Figure 3a). During the cycling performance measurement, after 200 cycles at 100 mA g^−1^, the PSC electrode exhibited a stable discharge capacity of 201 mAh g^−1^, much higher than CSC with a capacity of 133 mAh g^−1^ (Figure 3b). Similarly, PSC exhibited a better rate performance (Figure 3c).

**Figure 4:**
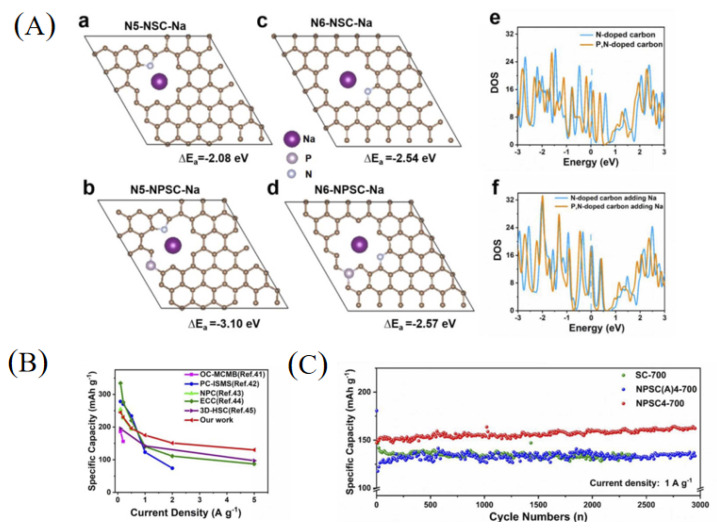
(**A**) Theoretical calculation between the pyrrolic-N and pyridinic-N structures and Na^+^ absorption (a–d: Single Na atom adsorption of N5-NSC, N5-NPSC, N6-NSC and N6-NPSC; e,f: The total density of states (TDOS) of N6-NSC, N6-NPSC and their structures after Na ^+^ adsorption.); (**B**) the comparison of rate performance with the reported anode materials; (**C**) long-term cycling performance [50] (Copyright 2022, Elsevier B.V).

Studies have shown that carbon frameworks with multi-atom doping can produce synergistic coupling effects between heteroatoms, thereby improving the electrochemical performance. Therefore, multi-atom doping in carbon materials was a good choice to achieve high-performance SIBs [17,26,49]. Recently, Zhao et al. [50] fabricated a soft carbon material with nitrogen and phosphorus co-doping as well as a nano-box morphology (NPSC4-700) using petroleum pitch as a starting material (Figure 3d). The obtained carbon material had a nitrogen content of 3.94 at% and phosphorus content of 1.14 at%. Through theoretical calculations (Figure 4A), the adsorption capacity of pyrrolic-N and pyridinic-N in NPSC increases, indicating that the nitrogen and phosphorus co-doping frameworks can contribute more adsorption capacity for sodium storage. As a result, the NPSC4-700 sample demonstrated a better rate performance for SIBs compared with some other carbons (Figure 4B). In addition, the NPSC4-700 sample also demonstrated an excellent cycling performance, showing a higher reversible capacity of 162 mAh g^−1^ after 3000 cycles at a current density of 1 A g^−1^ (Figure 4C).

**Figure 5:**
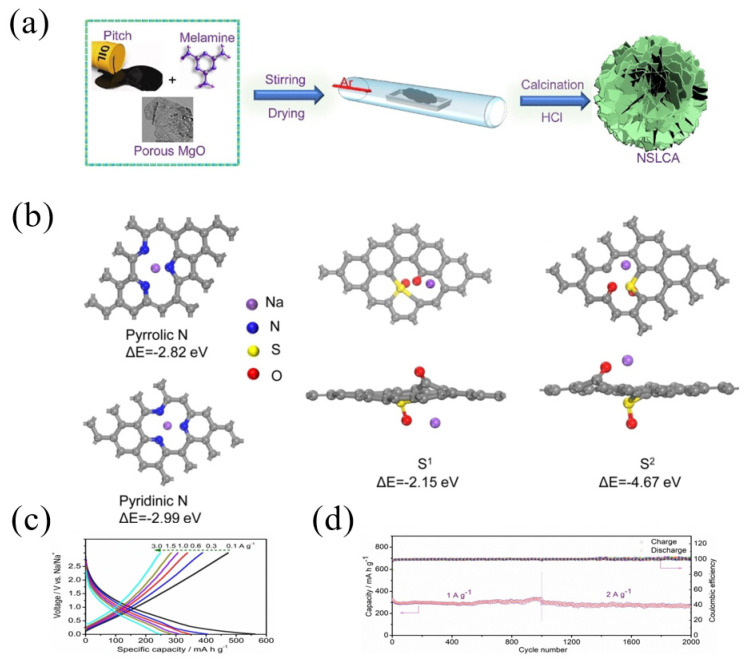
(**a**) Schematic illustration for the synthesis of ultrathin layered carbon with co-doped nitrogen and sulfur (NSLAC); (**b**) optimized structure and adsorbability of the pyrrolic N, pyridinic N, S at different locations; (**c**) charge–discharge profiles at various current densities and (**d**) cycling performance of NSLAC [51] (Copyright 2021, Elsevier B.V).

Furthermore, Sun et al. [51] fabricated ultrathin layered carbon with co-doped nitrogen and sulfur (NSLCA) through a template method using pitch as a carbon source, melamine as a nitrogen source and porous MgO as a template. The specific process was to mix pitch powder, the porous MgO template and melamine at 80 °C, and then the mixture was carbonized under Ar at 700 °C and the obtained NSLCA had a high nitrogen content of 9.5 wt.% and a sulfur content of 1.7 wt.% (Figure 5a). According to the DFT calculation, the binding energies were −2.82 eV and −2.99 eV for the adsorption of Na^+^ on two sites of nitrogen (pyrrolic N and pyridinic N), respectively, while the binding energies of the Na^+^ adsorbed on the S sites were −2.15 eV and −4.67 eV, respectively (Figure 5b), which demonstrated the improved sodium capacities after nitrogen and sulfur doping. As a result, the NSLCA electrode showed a better rate performance (Figure 5c), as well as a stable cycling performance; for instance, after 2000 cycles at high current densities of 1 and 2 A g^−1^, the NSLCA electrode delivered a high reversible specific discharge capacity of 290 and 270 mAh g^−1^, respectively (Figure 5d).

Recently, some great progress on pitch-based carbon with heteroatom doping for SIBs has been achieved. However, the uncertainty of the heteroatom content seriously affects the further development of pitch-based carbon materials. Therefore, how to optimize the reaction conditions and precisely control the content of heteroatoms in carbon is still a challenge.

### 2.3. Co-Carbonization

Pitch is a typical precursor for the synthesis of soft carbon, and soft carbon has a good electronic conductivity due to the high degree of graphitization; the microstructure of carbon is relatively orderly, and the interlayer spacing between carbon layers is small, resulting in the poor electrochemical performance of SIBs [52,53]. On the contrary, hard carbon has relatively large interlayer spacing and a relatively high Na storage capacity, but it is less conductive and the structure is highly disordered, resulting in a decreased rate performance and ICE of SIBs [54,55]. Therefore, combining hard carbon and soft carbon to form a carbon composite may be a feasible strategy to achieve efficient sodium storage [56].

**Figure 6:**
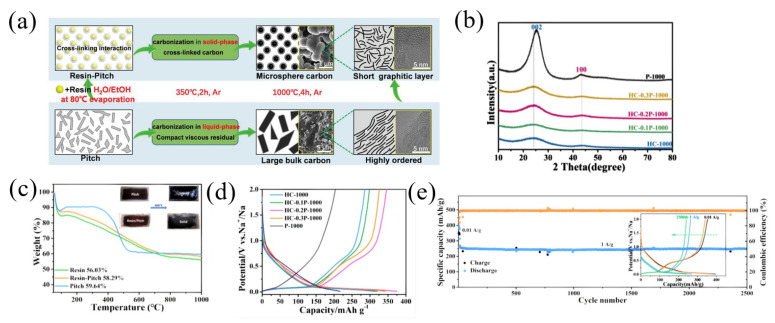
(**a**) The schematic diagram of co-carbonization of pitch and resin; (**b**) the XRD patterns of the obtained carbons; (**c**) TGA; (**d**) the second cycle charge–discharge profiles of the carbons; (**e**) long-term cycling performance measurement of sample HC-0.2P-1000 [58] (Copyright 2021, Wiley-VCH GmbH).

Phenolic resin is a typical carbon precursor for the synthesis of hard carbon [57]. In recent years, there have been many studies on the combination of phenolic resin and pitch to form a carbon composite. Using pitch and phenolic resin as the starting material, Li et al. [57] prepared pitch-derived amorphous carbon (PPAC), which delivered the highest reversible capacity of 284 mAh g^−1^ and the highest ICE of 88%. Similarly, using phenolic resin and pitch as the precursors, Yin et al. [58] fabricated resin-derived hard carbons (HCs) by adjusting the pitch weight ratio (10–30 wt.%) and pyrolysis temperature (800–1300 °C) (Figure 6a). As the proportion of pitch increased, the obtained carbon materials showed gradually obvious (002) and (100) peaks in XRD patterns. And according to the XRD result, the interlayer spacing decreased from 0.3757 nm to 0.3672 nm along with the increasing of pitch, demonstrating the enhanced graphitization of the carbon materials (Figure 6b). With the increase in temperature (800–1300 °C), the content of O decreased from 14.2% to 12.8%, and it mainly existed in the form of OH, C-O and C=O bonds, indicating that with the increase in temperature and the addition of pitch, the surface defects and polarization were reduced. The carbon yield of P-1000 (pure pitch), hard carbon (pure resin) and HC-0.2P-1000 (the mixture of pitch and resin) obtained after pyrolysis at 1000 °C was measured to be 59.64%, 56.03% and 58.29%, respectively, through a thermogravimetric analysis (TGA) (Figure 6c). When used as an anode for SIBs, the HC-0.2P-1000 and P-1000 samples showed a specific capacity (ICE) of 349.9 mAh g^−1^ (60.9%) and 206.5 mAh g^−1^ (52.4%), respectively (Figure 6d). In addition, HC-0.2P-1000 showed a good stability, and after 2500 cycles at a current density of 1 A g^−1^, HC-0.2P-1000 also delivered a reversible capacity of 249.3 mAh g^−1^ with a capacity retention of 94.5% (Figure 6e). 

**Figure 7:**
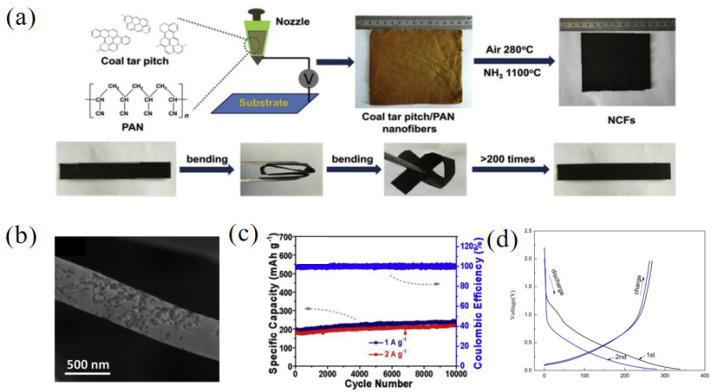
(**a**) Schematic illustration of the fabrication of pitch-derived carbon nanofibers (NCFs); (**b**) the SEM image of NCFs; (**c**) cycling performance of NCFs [59] (Copyright 2018, Elsevier Ltd.). (**d**) Charge/discharge profiles for the initial and second cycles of RGO/C800 sample [61] (Copyright 2018, Elsevier Ltd.).

Polyacrylonitrile (PAN) is also commonly used to compound with pitch to form carbon nanofibers. Carbon nanosheets have a good softness and high conductivity, so they have a high sodium ion storage capacity and great potential in the electronic field. Wang and co-workers [59] synthesized pitch-derived carbon nanofibers (NCFs) using coal tar pitch and PAN as starting materials through the treatment of NH_3_ (Figure 7a). Due to the engraving effect of NH_3_, the carbon nanofibers had a rough surface and a specific BET surface area of 546 m^2^ g^−1^ (Figure 7b). The results of an elemental analysis showed that with the addition of NH_3_, the N content of NCFs increased to 5.7%, and the contents of N-6 and N-5 also increased to 50.5% and 41.4%, respectively. The defects of N-6 and N-5 increased the storage of sodium ions and further improved the electrochemical performance of the samples. When used for SIBs, the obtained NCFs discharged at a current density of 0.1 A g^−1^ and exhibited a high reversible capacity of 345 mAh g^−1^. However, they delivered a low ICE of 53.4%. Moreover, NCFs had an excellent stability, showing a capacity of 235 and 217 mAh g^−1^ after 10000 cycles at 1 and 2 A g^−1^, respectively (Figure 7c). Reduced graphene oxide (RGO) has large layer spacing as well as an oxidic surface, which is suitable for improving the structure of pitch-derived carbon as well as final electrochemical properties [60]. For example, Yang et al. [61] prepared a composite called RGO/C800 using nonexpanded reduced graphene oxide and pitch through oil-phase treatment at a high temperature. With the increase in the pyrolysis temperature, the oxygen content of RGO/C800 decreased sharply from 32.73% to 4.51% compared with GO, indicating that most of the oxygen-containing functional groups were decomposed with the increase in the carbonization temperature. The results showed that the crystal structure of the RGO/C800 was amorphous and enlarged interlayer spacing was obtained. The nitrogen sorption isotherm measurement was performed on the sample RGO/C800 and the specific BET surface area was 3.00 m^2^ g^−1^, showing that with the addition of asphalt, the expansion of GO in the pyrolysis process is inhibited. When using RGO/C800 as an anode material in SIBs, the discharge/charge capacities were 338.9 mAh g^−1^/268.4 mAh g^−1^ and 233.3 mAh g^−1^/79.2 mAh g^−1^ for the initial and second cycles, respectively (Figure 7d). The ICE (79.2%) of RGO/C800 was much greater than that of pitch-based carbon without adding RGO (33.9%).

Due to the higher-degree graphitic structure and smaller interlayer spacing of pitch-derived carbon, a composite electrode with large layer spacing and a good conductivity can be formed by combining with hard carbon. The fabrication of a composite electrode can effectively increase the ICE and capacity. The ratio of hard and soft carbon and carbonization conditions have an important effect on the electrochemical properties of a carbon composite. Thus, the preparation of pitch-based composites with good electrochemical properties still needs in-depth exploration.

### 2.4. Pre-Oxidation

The pitch with direct pyrolysis at a high temperature generally goes through a liquid carbonization process, and the resulting product has a high graphitized structure and small spacing between layers. Plenty of oxygen-containing functional groups, introduced in the process of pre-oxidation of pitch, deter the melting and gathering of a carbon skeleton at high-temperature pyrolysis, and transform pitch to solid carbonization [62,63]. Therefore, pre-oxidation of pitch followed by carbonization effectively inhibits the degree of graphitization, thereby improving the energy storage performance of final pitch-based carbon materials [64].

**Figure 8:**
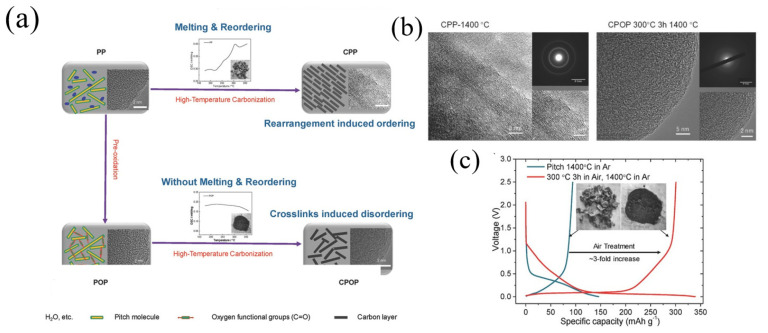
(**a**) Schematic illustration of CPP and CPOP preparation processes and structures; (**b**) the HRTEM and SAED images of CPP-1400 °C and CPOP-1400 °C; (**c**) the voltage curves of the first cycle and the second cycle of CPP-1400 °C and CPOP-1400 °C at a 0.1C rate and the digital images of CPP-1400 °C (**left**) and CPOP-1400 °C (**right**) samples [17] (Copyright 2018, WILEY-VCH Verlag GmbH & Co. KGaA, Weinheim).

Yuan et al. [65] fabricated a carbon material (OC-MCMB320) by combining air oxidation and pyrolysis. OC-MCMB320 delivered a reversible capacity of 286 mAh g^−1^ at a current density of 25 mA g^−1^. Using a facile pre-oxidation strategy, Lu et al. [17] fabricated disordered carbon materials (CPOP) with low-cost pitch (Figure 8a). Fourier transform infrared (FTIR) spectroscopy analysis and X-ray photoelectron spectra (XPS) results indicated that the oxygen was picked up and mainly presented in carbonyl (e.g., ester and anhydride). Compared to CPP1400 °C (1400 °C in Ar), CPOP-1400 °C (300 °C in Ar, 1400 °C in Ar) showed a relatively disordered graphene layer structure, and the corresponding SAED (Figure 8b) pattern was blurred, indicating that the pre-oxidation process could inhibit the graphitization processes. At a current density of 30 mA g^−1^, CPOP-1400 °C delivered a reversible capacity of 300.6 mAh g^−1^ with an ICE of 88.6%. In contrast, CPP1400 °C exhibited only a 94.0 mAh g^−1^ Na storage capacity with an ICE of 64.2% (Figure 8c). In addition, CPOP-1400 °C also revealed a good cycle stability, and the capacity could still maintain 93.1% after 200 cycles.

**Figure 9:**
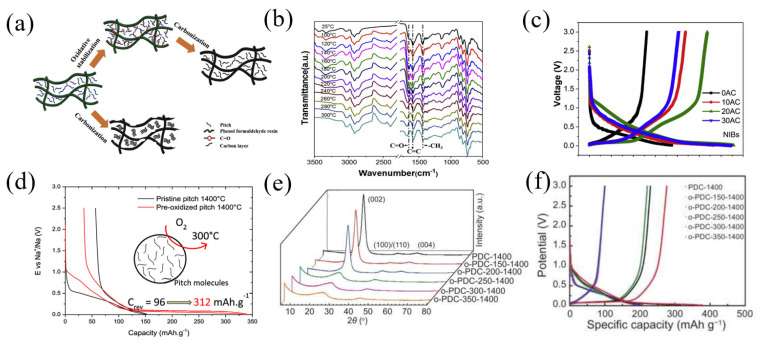
(**a**) Schematic illustration of the fabricating of amorphous carbon; (**b**) in situ FTIR spectra of a mixture of pitch and PR with temperature variation; (**c**) the 1st voltage profiles of four AC at 0.1 C; ref. [66] (Copyright 2019, Elsevier B.V). (**d**) Voltage profiles of HCPOP-ox12 at C/20 rate in the first cycle [67] (Copyright 2020 American Chemical Society). (**e**) XRD patterns of o-PDC-T-1400 series; (**f**) galvanostatic charge/discharge profiles of o-PDC-T-1400 [68] (Copyright 2021, Elsevier Limited).

Sun et al. [66] prepared a high-carbon-content (70%) amorphous carbon material controlled by the microstructure by mixing pre-oxidized pitch with phenol-formaldehyde resin and then carbonizing it. The mixture of pitch and phenolic resin was pre-oxidized at 220 °C with the different times of 0 h, 10 h, 20 h and 30 h in a heating furnace, then carbonized for 2 h at 1000 °C to obtain the amorphous carbons (0AC, 10AC, 20AC and 30AC) (Figure 9a). The FTIR result showed the vibration of the aromatic skeleton continuously strengthening, indicating that the introduction of oxygen led to pitch crosslinking with the phenol formaldehyde resin and formed network (Figure 9b). As the pre-oxidation time increased (0AC-20AC), the (002) peak in the XRD pattern became broader and shifted to a smaller diffraction angle, revealing that the d_002_ increased and the amorphous structure was more disordered. As shown in Figure 9c, all the voltage-capacity curves of carbon can be divided into two regions: a slant part above 0.1 V and a nearly straight-line platform at around 0.1 V. The sample 20AC exhibited the highest reversible capacity of 268.3 mAh g^−1^ at a constant current of 30 mA g^−1^. On the contrary, 0AC showed an almost all-slant voltage curve and only delivered a 130 mAh g^−1^ capacity. Moreover, 20AC had the highest ICE of 82% and 20AC delivered a specific capacity of 200 mAh g^−1^ and 106 mAh g^−1^ at 0.3 A g^−1^ and 1.2 A g^−1^, respectively, presenting an outstanding rate performance.

In order to research the influence of the atmosphere and duration on pitch pre-oxidation, Daher et al. [67] prepared hard carbons (HCPOP) by treating commercially available petroleum pitch in two steps, including pre-oxidation at 300 °C for different times (3, 12, 48, 72 and 200 h) and then connecting different atmospheres (ambient air, oxygen flow and air flow), followed by carbonization for 2 h at 1400 °C. The dates indicated that the pre-oxidation of pitch in air reached the limit of structural amorphization after 48–72 h. Under the oxygen atmosphere, more oxygen atoms were fixed into the pitch material and formed a network structure. HCPOP-ox12 (oxygen flow, 12 h) delivered the highest reversible capacity of 312 mAh g^−1^ at a current density of 96 mA g^−1^ (Figure 9d). Guo et al. [68] pre-oxidized pitch at a different oxidation temperature, followed by pyrolysis and synthesizing hard carbon materials with a temperature of 1400 °C under Ar atmospheres (o-PDC-T-1400, T represents the oxidation temperature). The XRD pattern showed that when the oxidation temperature was higher than 250 °C, the (002) peak in the XRD pattern of the sample became significantly wider and the graphite (004) peak disappeared (Figure 9e), indicating that the oxidation of pitch above 250 °C hindered the graphitization trend of carbon. With the rising temperature of oxidation, the I_D_/I_G_ value from Raman results elevated, and the result showed that the more defects added to the material, the degree of graphitization decreased. The o-PDC-350-1400 delivered the highest reversible capacity of 276.8 mAh g^−1^ at a current density of 100 mA g^−1^ with a high ICE of 73.38%. Compared with PDC-1400 (without pre-oxidation), the charging capacity was increased by about 1.8 times and the ICE was increased by 22% (Figure 9f).

In general, pre-oxidation of pitch can introduce oxygen atoms to form a crosslinking network between molecules, thereby effectively reducing the degree of graphitization during the pyrolysis process of pitch. The factors that affect pre-oxidation are the pre-oxidation temperature and pre-oxidation duration, as well as the carbonization temperature. These factors deserve further research and exploration when preparing pitch-based carbon materials using pre-oxidation methods.

## 3. Challenges and Perspectives

Pitch, a high-carbon-content and low-cost byproduct, mainly structured of polycyclic aromatic hydrocarbons, was considered to be a promising carbon precursor for the production of anode materials for SIBs. However, pitch-derived carbon materials usually present with a higher-degree graphitic structure and smaller interlayer spacing, which cannot satisfy the insertion of sodium. At present, not only modified pitch-based carbon itself can be modified by expanding the interlayer spacing and providing more active sites, but it also can be compounded with other high-capacity materials to improve the sodium storage performance of pitch-based carbon materials. Therefore, we need to measure between the manufacturing cost and high performance to select the optimal synthesis strategy.

A microstructure is a critical factor regarding the electrochemical properties of carbon anodes for SIBs. A porous structure provides rich channels for the easy diffusion of sodium ions, which effectively improves the electrochemical properties. At present, template carbonization is a common method to adjust the porous structure of pitch-based carbon. The addition of the template may lead to excessive etching, resulting in excessive defect sites, resulting in a sharp increase in the specific surface area, and an irreversible reaction during the sodium storage process, resulting in a lower ICE of porous carbon. Moreover, due to the addition of template reagents, the synthesis process of pitch-derived carbon is complex and the corresponding carbon yield is low, which affects the development of commercialization. Therefore, in a future commercial development process, how to select an appropriate template and simple strategy to accurately control the structure of pitch-based carbon on the basis of a guaranteed ICE value to achieve a balance between sodium storage active sites and the surface area as well as defects needs further research.

Heteroatom doping on a carbon material can provide more active sites for sodium ions and improve electron mobility, which has great potential for the sodium storage among pitch-derived carbon. Nonetheless, the synthesis of heteroatom-doped carbon usually requires heat treatment, but it will cause heteroatom loss during heat treatment, resulting in an uncertain heteroatom content. Therefore, it is still very challenging to accurately control the content of pitch-derived carbon heteroatoms. In the future, it is necessary to explore some new dopants and strategies without annealing to optimize the reaction conditions, accurately control the content of heteroatoms in carbon and realize the practical application of pitch coke and heteroatom doping.

The co-carbonization of pitch with an appropriate amount of a hard carbon precursor can not only greatly reduce the graphitization degree but also increase the conductivity of the final carbon-based material. Compared with a single component, the electrochemical properties of a hybrid carbon material are significantly enhanced. However, carbon precursors with different morphologies as well as multifunctional groups make it impossible to achieve the expected synergistic effect between pitch-based composite carbons, and most of the reagents used for crosslinking modification are costly, which makes them face challenges in large-scale practical applications. Therefore, in future research, it is necessary to fully understand carbon precursors with a strong synergistic effect with pitch-based carbon, further study the synergistic effect and mechanism between the components, optimize the carbonization conditions and combine the advantages of the two components.

Pre-oxidation can inhibit the liquid carbonization of pitch, reduce the degree of graphitization and increase the layer spacing. It is beneficial to increase a carbon yield, sodium storage capacity and relatively higher ICE. Simple air pre-oxidation is currently the most likely strategy for commercialization. Although the effects of the pre-oxidation temperature and pre-oxidation time on the microstructure of carbon materials and the electrochemical performance of carbon anodes for sodium ion batteries are worthy of attention, pre-oxidized pitch-derived carbon exhibits a low potential platform area of hard carbon on a charge–discharge curve, showing a higher sodium ion storage performance and high ICE. Therefore, simple air pre-oxidation is currently the strategy that is most likely to be commercialized.

## 4. Conclusions

This paper systematically reviews various tactics to transform pitch into pitch-based carbon with a suitable structure for SIBs, so as to reduce the degree of graphitization and provide more active sites for sodium, leading to an improved sodium storage performance. The electrochemical properties of various pitch-based carbon anodes reviewed in this paper are shown in Table 1. Despite challenges in the future, with the rapid development of pitch-derived carbon anodes, we can be optimistic that a more simple and environmentally friendly strategy will be developed in the future to achieve large-scale energy storage applications of pitch-based carbon materials.

## Figures and Tables

**Table 1:** The electrochemical performances of the samples prepared under four strategies.

Modification Strategy	Materials	Reversible Capacity	ICE	Cycle	Ref.
Porous structure adjustment	MSC	331 mAh g^−1^ at	45%	103 mAh g^−1^ after 3000	[31]
		30mA g^−1^		cycles at 50 mA g^−1^	
Porous structure adjustment	3DAC	280.1 mAh g^−1^ at	75%	188 mAh g^−1^ after 600	[32]
		30 mA g^−1^		cycles at 0.3 A g^−1^	
Porous structure adjustment	3DHSC-4	215 mAh g^−1^ at	60%	200.7 mAh g^−1^ after 120	[33]
		50 mA g^−1^		cycles at 50 mA g^−1^	
N-doped	PCNS1000	302 mAh g^−1^ at	66%	176 mAh g^−1^ after 1000	[41]
		0.1 A g^−1^		cycles at 0.5 A g^−1^	
N-doped	PMC-2	342 mAh g^−1^ at	84%	111 mAh g^−1^ after 1000	[42]
		37.2 mA g^−1^		cycles at 3 A g^−1^	
S-doped	SC800	482.8 mAh g^−1^ at	65.2%	103.7 mAh g^−1^ after 1000	[7]
		0.1 A g^−1^		cycles at 5 A g^−1^	
P-doped	PSC	251 mAh g^−1^ at	-	201 mAh g^−1^ after 200	[48]
		0.1 A g^−1^		cycles at 0.1 A g^−1^	
N, P co-doped	NPSC4-700	293 mAh g^−1^ at	35.45%	162 mAh g^−1^ after 3000	[50]
		50 mA g^−1^		cycles at 1 A g^−1^	
N, S co-doped	NSLCA	500 mAh g^−1^ at	-	270 mAh g^−1^ after 2000	[51]
		0.1 A g^−1^		cycles at 2 A g^−1^	
Co-carbonization	HC-0.2P-1000	349.9 mAh g^−1^ at	60.9%	249.3 mAh g^−1^ after 2500	[57]
		50 mA g^−1^		cycles at 1 A g^−1^	
Co-carbonization	NCFs	345 mAh g^−1^ at	53.4%	217 mAh g^−1^ after 10,000	[58]
		0.1 A g^−1^		cycles at 2 A g^−1^	
Co-carbonization	RGO/C800	268.4 mAh g^−1^ at	79.2%	239 mAh g^−1^ after 50	[59]
		20 mA g^−1^		cycles at 20 mA g^−1^	
Pre-oxidation	CPP1400 °C	300.6 mAh g^−1^ at	88.6%	279.4 mAh g^−1^ after 200	[64]
		30 mA g^−1^		cycles at 30 mA g^−1^	
Pre-oxidation	20AC	268.3 mAh g^−1^ at	82%	190 mAh g^−1^ after 200	[65]
		30 mA g^−1^		cycles at 0.3 A g^−1^	
Pre-oxidation	HCPOP-ox12	312 mAh g^−1^ at	86%	-	[17]
		96 mA g^−1^			
Pre-oxidation	o-PDC-350-1400	276.8 mAh g^−1^ at	73.38%	170.2 mAh g^−1^ after 200	[66]
		0.1 A g^−1^		cycles at 30 mA g^−1^	
